# Senescent skeletal muscle fibroadipogenic progenitors recruit and promote M2 polarization of macrophages

**DOI:** 10.1111/acel.14069

**Published:** 2023-12-19

**Authors:** Xu Zhang, Yan Er Ng, Lucas C. S. Chini, Amanda A. Heeren, Thomas A. White, Hao Li, Haojie Huang, Madison L. Doolittle, Sundeep Khosla, Nathan K. LeBrasseur

**Affiliations:** ^1^ Robert and Arlene Kogod Center on Aging Mayo Clinic Rochester Minnesota USA; ^2^ Department of Biochemistry and Molecular Biology Mayo Clinic Rochester Minnesota USA; ^3^ Paul F. Glenn Center for Biology of Aging Research at Mayo Clinic Rochester Minnesota USA; ^4^ Division of Endocrinology Mayo Clinic Rochester Minnesota USA; ^5^ Department of Physical Medicine and Rehabilitation Mayo Clinic Rochester Minnesota USA

**Keywords:** cellular senescence, fibroadipogenic progenitors (FAPs), macrophages, migration, polarization

## Abstract

Senescent cells compromise tissue structure and function in older organisms. We recently identified senescent fibroadipogenic progenitors (FAPs) with activated chemokine signaling pathways in the skeletal muscle of old mice, and hypothesized these cells may contribute to the age‐associated accumulation of immune cells in skeletal muscle. In this study, through cell–cell communication analysis of skeletal muscle single‐cell RNA‐sequencing data, we identified unique interactions between senescent FAPs and macrophages, including those mediated by *Ccl2* and *Spp1*. Using mouse primary FAPs in vitro, we verified increased expression of *Ccl2* and *Spp1* and secretion of their respective proteins in the context of both irradiation‐ and etoposide‐induced senescence. Compared to non‐senescent FAPs, the medium of senescent FAPs markedly increased the recruitment of macrophages in an in vitro migration assay, an effect that was mitigated by preincubation with antibodies to either CCL2 or osteopontin (encoded by *Spp1*). Further studies demonstrated that the secretome of senescent FAPs promotes polarization of macrophages toward an M2 subtype. These data suggest the unique secretome of senescent FAPs may compromise skeletal muscle homeostasis by recruiting and directing the behavior of macrophages.

## Graphical abstract

Through bioinformatic analysis of single‐cell RNA‐sequencing data, we identified unique communication patterns between senescent fibroadipogenic progenitors (FAPs) and macrophages in skeletal muscle of old mice. Using an in vitro model, we demonstrate senescent skeletal muscle‐derived FAPs effectively recruit macrophages, in part, by secretion of the chemokine (C‐X‐C motif) ligand 2 (CCL2) and osteopontin (OPN). Finally, we show the robust secretome of senescent FAPs promotes M2 polarization of macrophages, the subtype evident in skeletal muscle of older mice and humans.

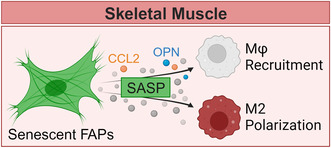

**Abbreviations**
BMDM: Bone Marrow Derived Macrophages
BMP: Bone Morphogenetic Protein
CCL: The Chemokine (C‐C motif) Ligand
CXCL: The chemokine (C‐X‐C motif) Ligand
FAPs: Fibroadipogenic Progenitors
FGF: Fibroblast Growth Factors
GSEA: Gene Set Enrichment Analysis
IGF: Insulin‐like Growth Factor
MK: midkine
ncWNT: non‐canonical WNT
OPN: Osteopontin
SASP: Senescence‐Associated Secretory Phenotype
SA‐β‐Gal: Senescence‐Associated Beta‐Galactosidase
scRNA‐seq: Single‐Cell RNA‐Sequencing

## 1 INTRODUCTION

Fibroadipogenic progenitors (FAPs) are dynamic cells that reside within the interstitial space of skeletal muscle. As their name implies, FAPs can differentiate into fibroblasts and adipocytes, and as a result, contribute to skeletal muscle fibrosis and lipid accumulation. FAPs can also influence the behavior of other resident cells in skeletal muscle, including satellite cells and myofibers, and facilitate the recruitment of immune cells through various signaling molecules (Biferali et al., 2019). These actions are important for skeletal muscle homeostasis, and are particularly evident in the context of injury. Correspondingly, depletion of FAPs in regenerating skeletal muscle compromises expansion of both satellite cells and immune cells (Wosczyna et al., 2019). These data highlight the critical role of FAPs in maintaining skeletal muscle health and function through cell–cell communication.

Senescence is a cell fate in response to diverse forms of stress, characterized by stable growth arrest, altered morphology, chromatin reorganization, organelle dysfunction, and secretion of biologically active molecules, termed the senescence‐associated secretory phenotype (SASP) (Coppe et al., 2010). While senescence is a conserved mechanism to counter replication of damaged preneoplastic cells (Collado & Serrano, 2010), senescent cells accumulate with age and contribute to the dysfunction of multiple tissues, in part, through their SASP (Khosla et al., 2020). In a recent study, we identified a subpopulation of FAPs in skeletal muscle of old mice with core features of cellular senescence, including high expression of the cyclin‐dependent kinase inhibitor p16^ink4a^ along with genes involved in chemokine signaling and cytokine–cytokine receptor interactions (Zhang et al., 2022). Although the significance of the altered transcriptional profile of senescent compared to non‐senescent FAPs in skeletal muscle has not been examined, its characteristics suggest it may influence their communication with other cells.

Across tissues, immune cells are primary recipients of signals from senescent cells. In the context of health, immune cells facilitate the removal of senescent cells, while in disease they can instead amplify the proinflammatory SASP (Behmoaras & Gil, 2021; Sturmlechner et al., 2021). Macrophages are a key component of the innate immune response in tissues such as skeletal muscle and play an important role in the maintenance of tissue homeostasis, and the response to infection and injury (Sousa et al., 2023). Macrophages can differentiate into different subgroups with specific and even opposing biological functions (Krasniewski et al., 2022). When activated by IFNγ or LPS, macrophages undergo M1 polarization and produce inflammatory cytokines and nitric oxide to eliminate bacteria, viruses, or dysfunctional cells. When activated with IL‐4 and IL‐13, macrophages undergo M2 polarization and exhibit more of an anti‐inflammatory phenotype, but, interestingly, can also promote fibrosis (Braga et al., 2015). In fact, in skeletal muscle of older mice and humans, macrophages are shifted in favor of the M2 subtype, and appear to contribute to age‐related collagen deposition (Cui et al., 2019; Wang et al., 2015). Given the interplay between FAPs and macrophages, the susceptibility of FAPs to senescence, and the marked shift in the transcriptional profile of FAPs as a consequence of senescence, it is plausible that senescent FAPs differentially recruit and influence the phenotype of macrophages.

To test this hypothesis, we first analyzed cell–cell communication patterns in skeletal muscle of young and old mice using single‐cell RNA‐sequencing (scRNA‐seq) data. This revealed extensive but distinct cross‐talk between non‐senescent compared to senescent FAPs and macrophages. Next, we examined candidate SASP factors mediating FAP‐macrophage interactions and then studied the influence of the SASP on markers of macrophage polarization using an in vitro model consisting of mouse primary FAPs and mouse macrophages.

## 2 METHODS

### 2.1 Cell–cell communication analysis using CellChat

Single‐cell RNA‐seq data of FAPs and macrophages within mouse skeletal muscle, including data from three 6‐month‐old mice and three 24‐month‐old mice, were extracted from GSE172410. FAPs were separated into non‐senescent and senescent populations as published (Zhang et al., 2022). The Seurat object containing the non‐senescent FAPs, senescent FAPs, and macrophages were merged, filtered, normalized, and scaled using Seurat (V3.1.3), and the cell–cell communication analysis was performed using Cellchat (v1.0.0) following the standard workflow (Jin et al., 2021). Details can be found in their tutorial at https://github.com/sqjin/CellChat.

### 2.2 Mouse information and cell culture

The use of male and female C57BL/6 mice for cell isolation was approved by the Mayo Clinic Institutional Animal Care and Use Committee. FAPs were isolated from 3‐ to 6‐month‐old mouse skeletal muscle (Quadriceps, Gastrocnemius, and tibialis anterior muscles) using magnetic‐activated cell sorting for the CD45‐/CD31‐/α7‐integrin‐/SCA‐1+ population (Zhang et al., 2022). FAPs were cultured in Dulbecco's Modified Eagle Medium (DMEM, Gibco) supplemented with 20% fetal bovine serum (FBS), 1% penicillin–streptomycin‐glutamine, and 10 ng/mL recombinant human bFGF at 37°C with 95% air, 5% CO_2_ and 3% O_2_. To induce senescent FAPs in vitro, cells were treated with either 20μM of etoposide for 48 h and 5 more days in a normal medium, or with 10 Gy of X‐ray and then maintained for 21 days. RAW264.7 macrophages were cultured in DMEM supplemented with 10% FBS and 1% penicillin–streptomycin‐glutamine at 37°C with 95% air and 5% CO_2_. BMDM cells were isolated from bone marrow of 3‐ to 6‐month‐old mouse as previously described with some modifications (Zhang et al., 2017). Briefly, bone marrow was isolated from femurs and tibias, treated with red blood cell lysis buffer, filtered through a 70 μm strainer, and then plated in DMEM supplemented with 10% FBS, 1% penicillin–streptomycin‐glutamine, and 25 ng/mL M‐CSF at 37°C with 95% air, 5% CO_2_. Five days later, cells were split for treatment with conditioned medium from non‐senescent or senescent FAPs.

### 2.3 Senescence‐associated β‐galactosidase (SA‐β‐gal) staining

Senescence beta‐galactosidase staining kit (Cell Signaling Technology, Cat # 9860S) was used for the beta‐galactosidase staining.

### 2.4 RNA isolation and quantitative real‐time PCR

RNA was extracted using Trizol method, reverse transcribed to cDNA, and analyzed by quantitative real‐time PCR using Taqman low ROX master mix. Primers and probes for qPCR are listed in the supplementary key resource Table S1.

### 2.5 RNA sequencing and data analysis

For RNAseq, libraries were prepared using TruSeq Stranded mRNA Sample Prep kit and sequenced using the Illumina cBot and HiSeq 3000/4000 PE Cluster kit on an Illumina HiSeq 4000. Differentially expressed genes were analyzed using EdgeR (V3.10) in R (V3.6.0). Gene Set Enrichment Analysis was performed using software GSEA (v.4.0.3).

### 2.6 Magpix assay

Conditioned medium was collected from either non‐senescent or senescent FAPs in DMEM 4.5 g/l glucose supplemented with 10% FBS and 1% penicillin–streptomycin‐glutamine at 37°C at 3% O_2_ incubator for 24 h. Conditioned medium was filtered through 0.22 μm filter before analysis, and the cell number was quantified using an automated cell counter (Biorad) for normalization. The concentrations of GDF‐15, TNFRI, TNFRII, IL‐6, IL‐10, ICAM‐1, PAI‐1, Eotaxin, MCP‐1, MMP‐2, and OPN in 50 μL of conditioned medium were quantified using multiplex magnetic bead immunoassays (R&D Systems).

### 2.7 Macrophage migration and polarization assay

Macrophage migration was assessed using a transwell assay in a 24‐well plate with polycarbonate membrane inserts with 8.0 μm pores (Corning). Briefly, 1 x 10^5^ of RAW264.7 cells were seeded in the insert (upper chamber) in 100 μL of medium with 0.1% BSA. Lower chambers were then filled with 600 μL of either conditioned medium, or medium with 0.1% BSA as a negative control. For some wells, antibodies against OPN (100 ng/mL) or CCL2 (100 ng/mL) were added into the conditioned medium. After culture for 24 h, the migrated cells on the lower surface of the membrane were stained with DAPI (Invitrogen) and imaged using an EVOS microscope (Thermo) and quantified using Fiji.

Macrophage polarization was assessed by culturing the RAW264.7 or BMDM in the conditioned medium for 24 h. Medium containing LPS and IFN‐gamma or IL‐4 and IL‐13 were used for M1/M2 control. After 24 h of treatment, cells were scraped off the dish and collected for RNA isolation or staining and Flow Cytometry analysis.

### 2.8 Flow cytometry analysis

Cells were harvested by scraping from dishes, and a total of 1 × 10^6^ cells were suspended in 500 μL of 5% FBS/PBS solution, and incubated with APC anti‐mouse CD68 antibody (Biolegend Cat# 137008), PE anti‐mouse CD80 antibody (Biolegend Cat# 104708), FITC anti‐mouse CD206 antibody (Biolegend Cat# 141704) under the manufacturer recommended concentration for 1 h. After being washed with PBS solution 3 times, cells were loaded into the flow cytometer (BD FACSCanto, BD biosciences) and data were analyzed with FlowJo software (v10.8.1). More details are listed in the supplementary key resource Table S1.

### 2.9 Statistical analysis

Statistical analyses were carried out using GraphPad Prism 8, and a *p* < 0.05 was considered statistically significant. For comparisons between 2 groups, unpaired *t*‐test was used. For comparisons between multiple groups, a one‐way ANOVA was used. All data are expressed as the mean ± SEM (standard error of the mean).

## 3 RESULTS

### 3.1 Senescence alters FAP to macrophage communication

**FIGURE 1:**
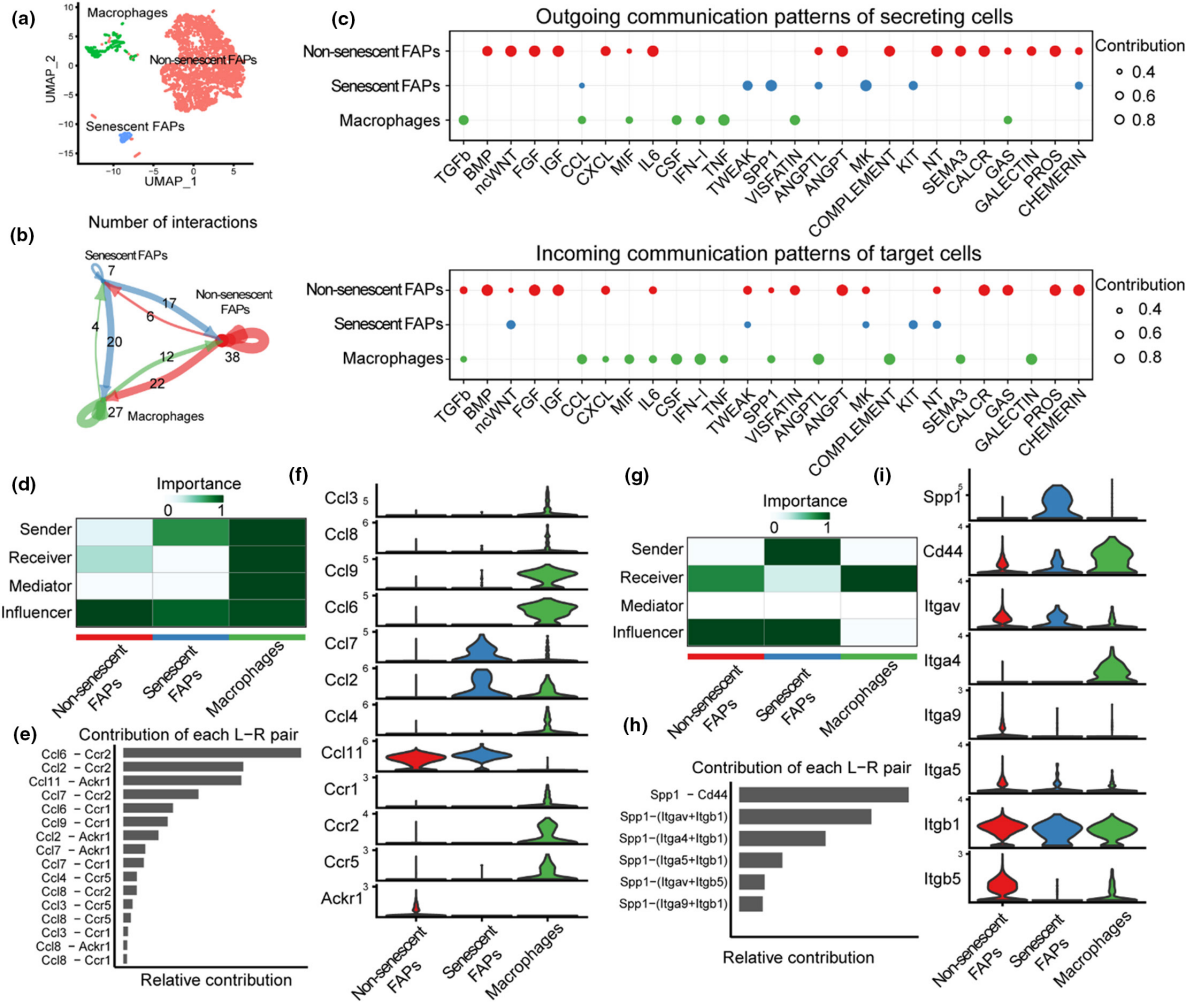
Senescence alters FAP autocrine and paracrine signaling in skeletal muscle. (a) Integration of scRNA‐seq data from non‐senescent FAPs, senescent FAPs, and macrophages. (b) CellChat analysis revealed an extensive intercellular communication network between non‐senescent and senescent FAPs and macrophages. (c) Dot plot showing significant outgoing (sender) and incoming (receiver) pathways of different cell subpopulations. (d,g) Significant interactions in CCL (d) and SPP1 (g) signaling pathways between senescent FAPs and macrophages. Heatmap showing the dominant senders and receivers in the network. (e,h) Contribution of different ligand and receptors in the CCL (e) and SPP1 (h) pathway. (f,i) Violin plot showing the gene expression of the contributing ligands and receptors in the CCL2 (f) and SPP1 (h) signaling pathway.

We recently reported that a subpopulation of FAPs in the skeletal muscle of old (24 months of age) mice exhibit hallmarks of cellular senescence, including increased expression of p16 and gene sets associated with chemokine signaling and cytokine–cytokine receptor interactions along with elevated markers of DNA damage, and chromatin reorganization (Zhang et al., 2022). To study the influence of senescence on cell–cell communication patterns, we analyzed scRNA‐seq data using CellChat (Jin et al., 2021). Leveraging the expression profiles of ligands, receptors, and their associated cofactors across various cell populations, CellChat can predict the primary signaling inputs and outputs in cells, as well as how these cells and signals orchestrate their functions in a coordinated manner (Jin et al., 2021). Extensive yet distinct ligand and receptor pairs, and autocrine and paracrine communication patterns were revealed between non‐senescent FAPs and senescent FAPs (Figure 1a–c and Figure S1).

Non‐senescent FAPs exhibited a multitude of outgoing signals, including BMP, ncWNT, FGF, IGF, CXCL, and galectin (Figure 1c). In addition to being the senders, non‐senescent FAPs were also the receivers of many of these signals (e.g., BMP, FGF, and galectin), consistent with autocrine signaling. To a lesser extent, non‐senescent FAPs signaled to macrophages through outgoing communication patterns such as complement, SMEA3, and galectin, indicative of paracrine signaling. Interestingly, many of the outgoing signals observed in non‐senescent FAPs were absent in senescent FAPs, as were several incoming autocrine signals, including BMP, ncWNT, FGF, IGF, CXCL, complement, SEMA3, and galectin. Instead, senescent FAPs were the unique senders of chemokine ligands (CCLs), osteopontin (SPP1), midkine (MK), and TWEAK (Figure 1c).

CCLs and SPP1 are chemokines commonly associated with the SASP. Accordingly, macrophages were the receivers of CCL incoming communication patterns (Figure 1d). Further analysis revealed *Ccl6*, *Ccl2*, *Ccl11*, and *Ccl7* were the major ligands contributing to this interaction, while *Ccr2* and *Ackr1* were the major receptors (Figure 1e). Notably, *Ccl2*, *Ccl7*, and *Ccl11* were uniquely or most highly expressed in senescent FAPs while their major receptor *Ccr2* was only expressed in macrophages (Figure 1f).

A similar cell–cell communication pattern was observed for the SPP1 pathway (Figure 1g), as further investigation of scRNA‐seq data demonstrated that the receptor of this senescent FAP‐derived ligand, *Cd44*, was highly expressed in macrophages (Figure 1h,i). Collectively, these data suggest that cellular senescence fundamentally alters outgoing communication patterns from FAPs by inducing the loss of autocrine signals and the gain of paracrine chemokine signals, particularly to macrophages, in the skeletal muscle of old mice.

### 3.2 An in vitro model of senescent FAPs

**FIGURE 2:**
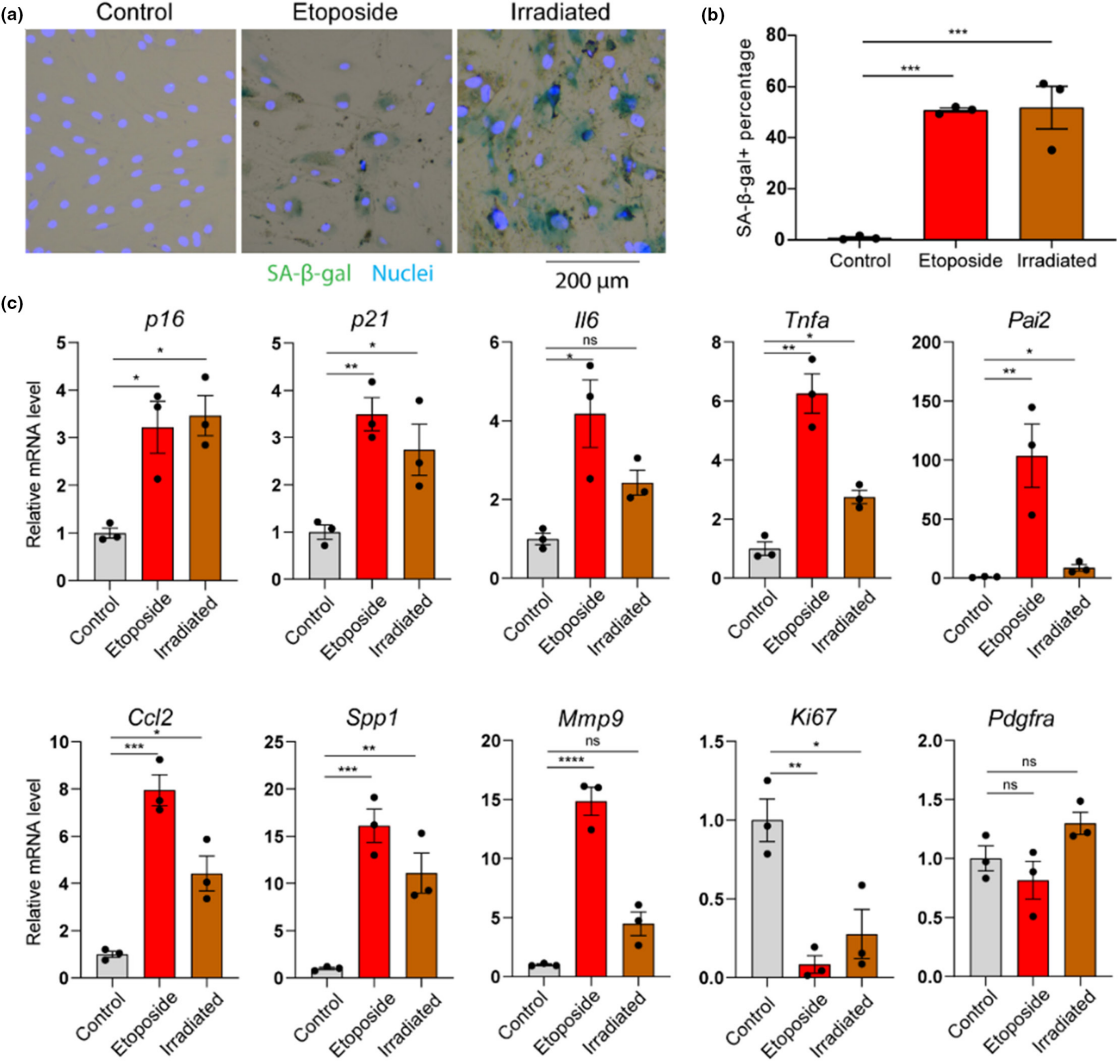
Features of an in vitro model of FAP senescence. (a) Representative SA‐β‐gal staining images of control, etoposide challenged, and irradiated FAPs. (b) Quantification of the SA‐β‐gal positive cells. (c) Gene expression of cell type, cyclin‐dependent kinase inhibitors, SASP components, and a proliferation marker quantified by qPCR. *N* = 3 per group, one‐way analysis of variance (ANOVA). Error bars represent SEM. *, **, ***,**** denote *p* < 0.05, 0.01, 0.001, and 0.0001, respectively.

To further explore the impact of senescence on cell–cell communication, we developed an in vitro model using mouse primary FAPs. FAPs were isolated from skeletal muscle based on surface markers CD31‐CD45‐Integrin α‐7‐SCA1+. Senescence was induced by etoposide or X‐ray irradiation. Consistent with a senescent phenotype, cells challenged by both stressors exhibited significantly greater size and senescence‐associated beta‐galactosidase (SA‐β‐Gal) staining relative to control cells (Figure 2a,b). Moreover, analysis by qPCR demonstrated increased expression of the cyclin‐dependent kinase inhibitors *p16* and *p21* and several cytokine and chemokine components of the SASP, including *Ccl2* and *Spp1*, and decreased expression of the proliferation marker Ki67, in etoposide and irradiated FAPs compared to control FAPs, consistent with cellular senescence (Figure 2c).

### 3.3 The transcriptome of etoposide‐induced senescent FAPs recapitulates core features of in vivo senescent FAPs

**FIGURE 3:**
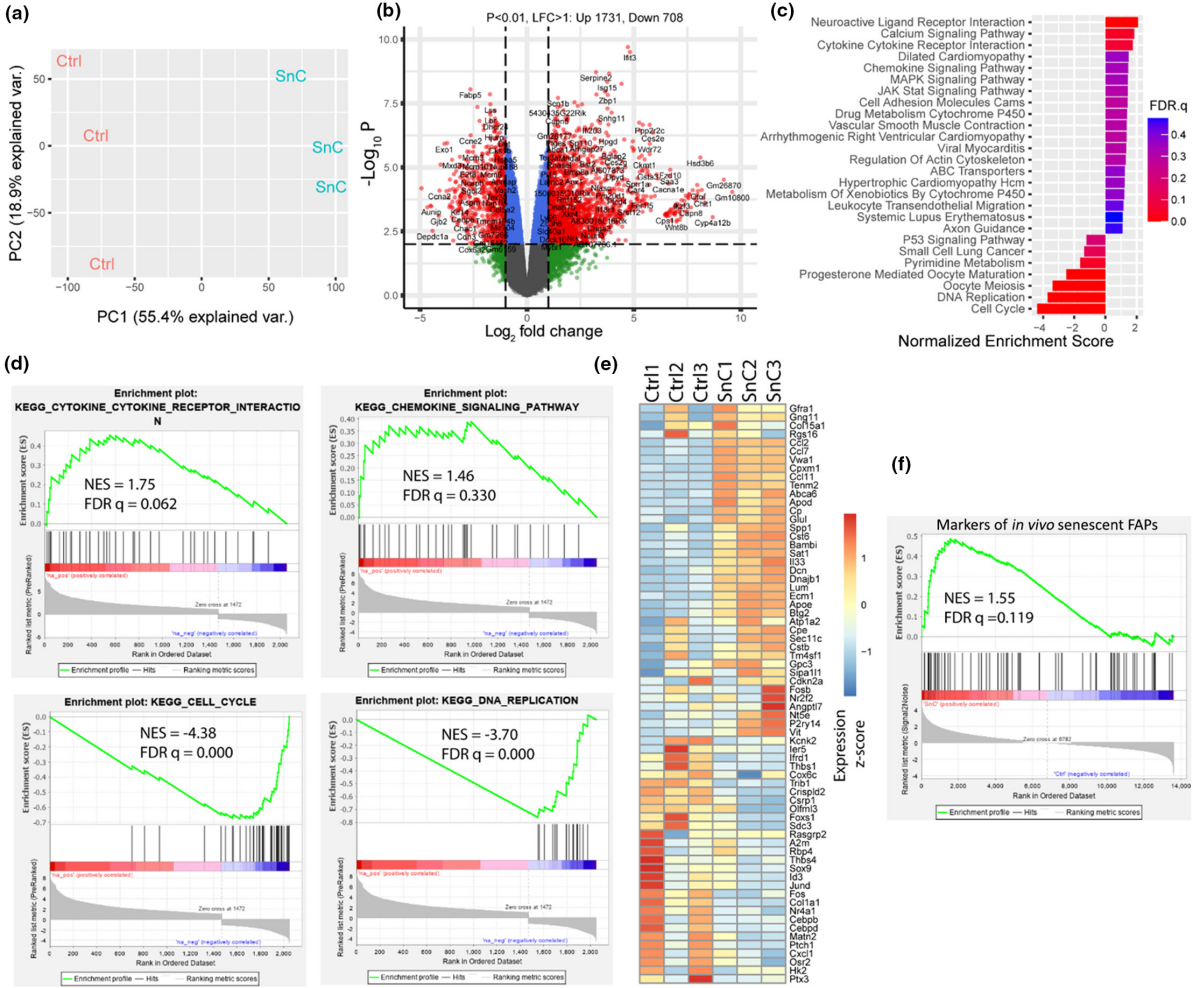
Core features of the senescent FAP transcriptome in vivo are replicated in vitro. (a) PCA plot of the transcriptomes of the six samples analyzed by RNAseq. (b) Volcano plot showing the differently expressed genes of senescent compared to control FAPs, with a log fold change (LFC) exceeding 1 and a p‐value less than 0.01. (c) Enriched KEGG pathways in senescent compared to control FAPs using the DEGs. (d) GSEA enrichment plot of the cytokine–cytokine receptor interaction pathways, chemokine signaling pathway, cell cycle, and DNA replication pathways. (e) Normalized expression level of markers for in vivo senescent FAPs, a subpopulation of FAPs positive for p16 and senescence‐related gene sets as reported (Zhang et al., 2022), in control FAPs and etoposide‐induced senescent FAPs. (f) GSEA enrichment plot of etoposide‐induced senescent FAPs compared to the control FAPs. Normalized enrichment score (NES) and FDR q‐value was calculated in GSEA software.

To further define the senescence phenotype induced in vitro, we performed bulk RNA‐seq on both control (non‐senescent) and etoposide‐induced senescent FAPs. Principal components analysis exhibited stark differences in the transcriptional profiles of the cells (Figure 3a), evidenced by 1731 activated and 708 repressed genes in senescent compared to control FAPs (Figure 3b). Gene set enrichment analysis (GSEA) showed upregulation of cytokine–cytokine receptor interactions, chemokine signaling, and the MAPK signaling pathway and downregulation of DNA replication and cell cycle pathways (Figure 3c,d), which strongly align with conserved features of the senescence program. Correspondingly, we observed considerable agreement between the gene sets and their components enriched in senescent FAPs in vitro, and those we identified by GSEA of scRNA‐seq data from senescent FAPs in vivo (Zhang et al., 2022), including the chemokines *Ccl2*, *Ccl7*, and *Spp1* (Figure 3e,f). These data suggest that the in vitro model of senescent FAPs recapitulates core features of senescence driven in vivo by aging.

### 3.4 Senescent FAPs are programmed to effectively recruit macrophages

**FIGURE 4:**
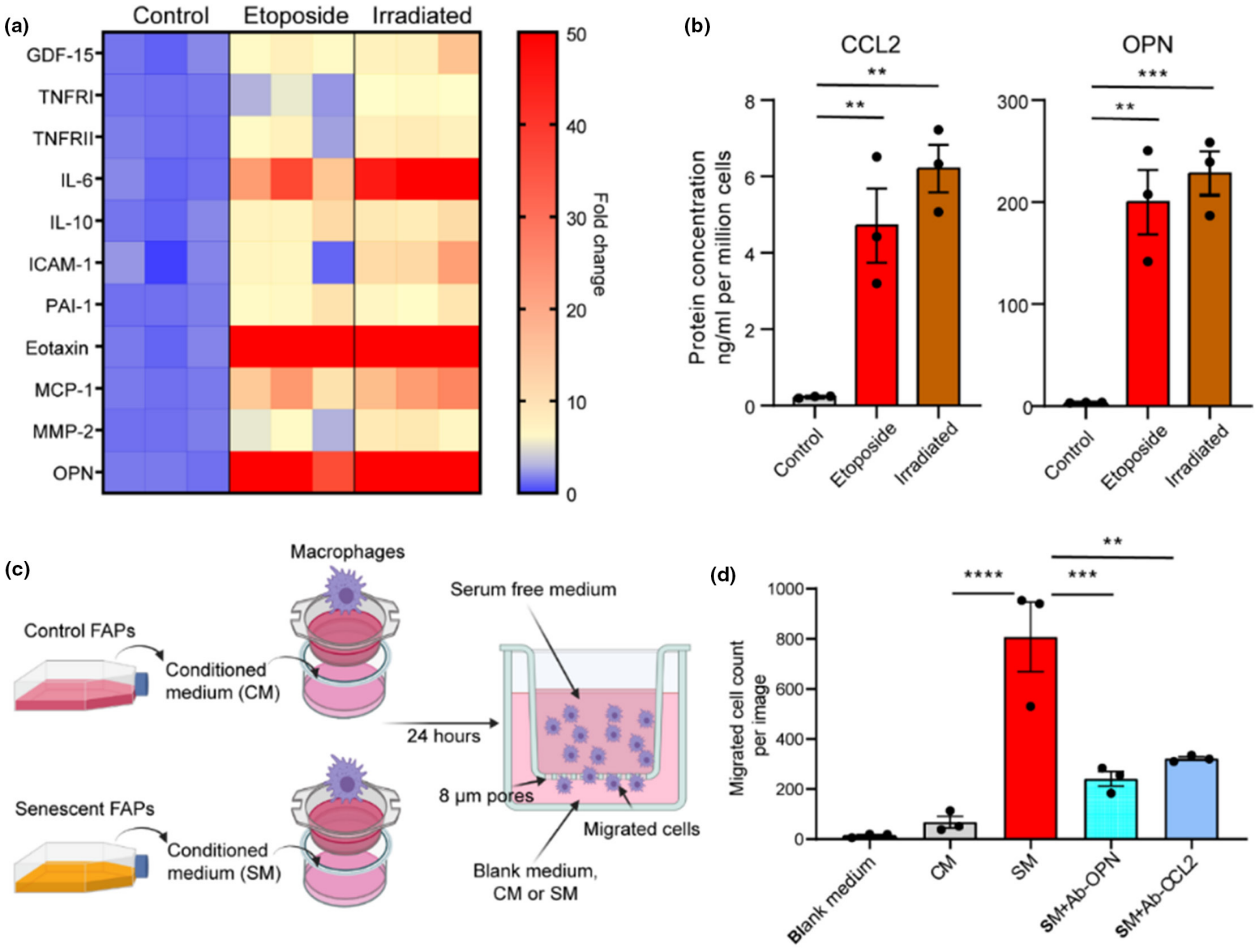
Senescent FAPs actively recruit macrophages in vitro. (a) Compared to non‐senescent (control) cells, etoposide‐ and irradiation‐induced senescent FAPs robustly secrete diverse proteins into their medium. (b) Protein concentration of CCL2 and OPN in the conditioned medium of control FAPs and etoposide‐ and irradiation‐induced senescent FAPs. (c) Schematic of the macrophage migration experiment, using control and senescent FAPs. (d) Quantification of the macrophages recruited by blank (cell‐free), non‐senescent control (CM), or senescent (SM) conditioned medium without and with antibodies to OPN (+Ab‐OPN) and CCL2 (+Ab‐CCL2). *N* = 3 per group, one‐way analysis of variance (ANOVA). Error bars represent SEM. **, ***, and **** denote *p* < 0.01, 0.001, and 0.0001, respectively.

We hypothesized that senescence‐induced changes in outgoing communication patterns and the transcriptional profile of FAPs both in vivo and in vitro affect interactions with macrophages. Thus, we first corroborated transcript data consistent with upregulation of the SASP by quantifying the abundance of several signaling proteins in the medium of non‐senescent control and senescent FAPs. Both etoposide‐ and X‐ray‐induced senescent FAPs exhibited a robust and diverse SASP, inclusive of CCL2 and osteopontin (OPN, the protein product of *Spp1*) (Figure 4a). The protein concentration of CCL2 was approximately 20‐fold higher while OPN was approximately 50‐fold higher in the medium of senescent compared to control FAPs (Figure 4b). We then used conditioned medium from control and senescent FAPs in an in vitro macrophage migration assay, using an established mouse macrophage RAW264.7 cell line (Figure 4c). Senescent cell conditioned medium stimulated a 12‐fold greater increase in the migration of macrophages than control medium. Notably, preincubation of senescence medium with neutralization antibodies against either OPN or CCL2 significantly attenuated the number of macrophages recruited (Figure 4d). These data indicate senescence markedly alters the molecular phenotype and secretome of FAPs in a manner that strongly promotes immune cell recruitment, in part, through CCL2 and OPN.

### 3.5 Senescent FAPs promote macrophage polarization toward M2

**FIGURE 5:**
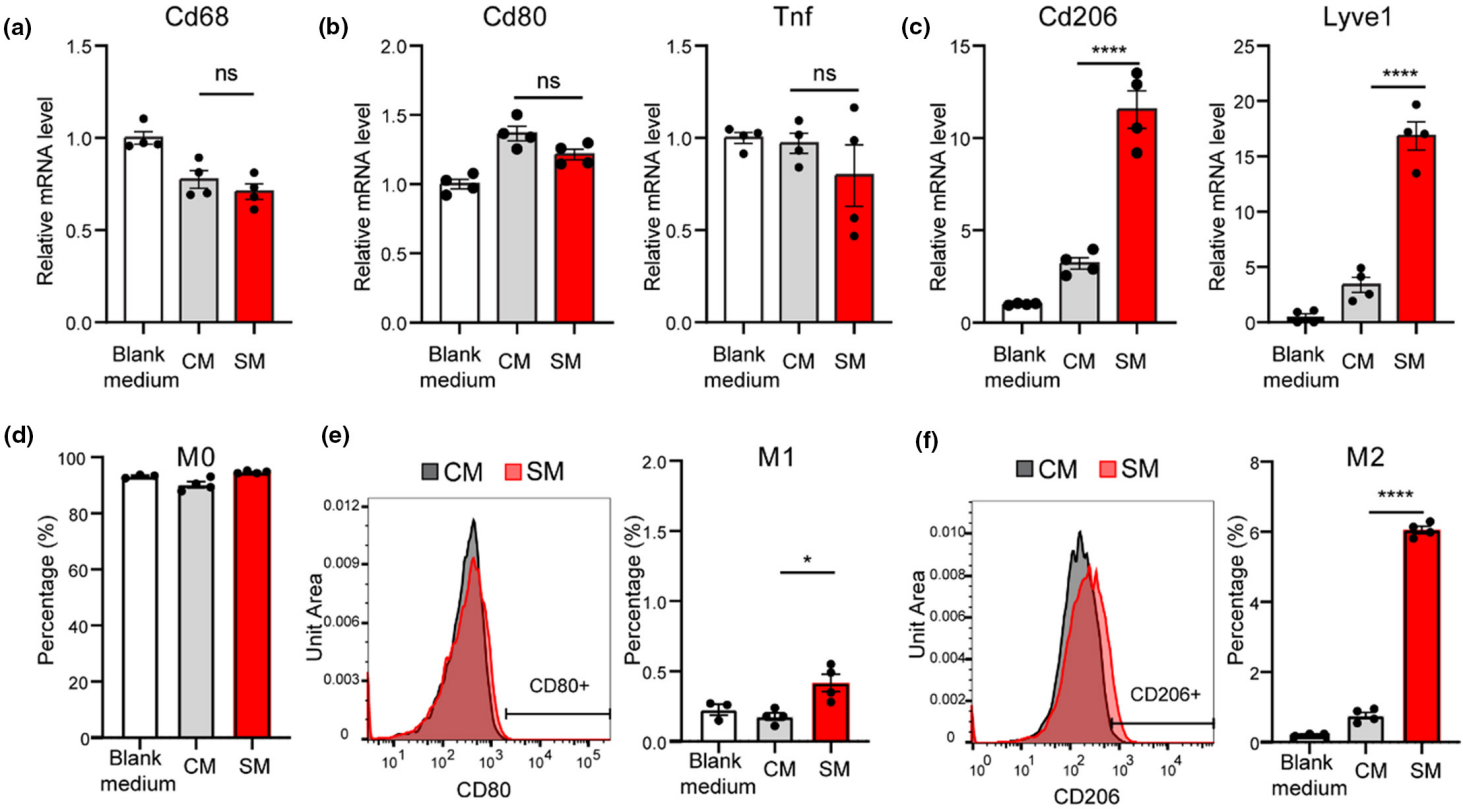
Senescent FAPs promote macrophage polarization toward M2. (a–c) Gene expression of M0 (a) M1 (b) and M2 (c) macrophage markers in blank (cell‐free) medium, or medium with non‐senescent (CM), or senescent (SM) conditioned medium. (d–f) Relative cell subtype abundance quantified with flow cytometry for M0 (d), M1 (e), and M2 (f) macrophages. *N* = 4 per group, one‐way analysis of variance (ANOVA). Error bars represent SEM. * and **** denote *p* < 0.05 and 0.0001, respectively.

Next, we studied how senescence‐driven changes in the FAP secretome influence the polarization of macrophages, using mouse bone marrow derived macrophages (BMDM). BMDM were cultured in growth (blank) medium or conditioned medium from non‐senescent or senescent FAPs for 24 h. Medium from neither control nor senescent FAPs affected BMDM expression of the pan M0 macrophage marker *Cd68* or M1 macrophage markers *Cd80* and *Tnfα* compared to blank medium, as assessed by qPCR (Figure 5a,b). However, compared to control medium, senescent medium significantly increased the expression of the traditional M2 macrophage marker *Cd206*, as well as *Lyve1* (Figure 5c), which was recently identified as a novel M2 marker (Childs et al., 2021; Krasniewski et al., 2022).

To corroborate these data, we again incubated BMDM in blank medium or conditioned medium from non‐senescent or senescent cells, but then labelled BMDM with antibodies for surface markers for M0 (CD68), M1 (CD80), and M2 (CD206), and quantified the number of cells in each subset using flow cytometry. Compared to blank medium, neither control nor senescent medium altered the percentage of M0 macrophages (Figure 5d), while senescence medium modestly yet significantly increased the percentage of M1 macrophages. Most strikingly and consistent with analysis by qPCR, senescence medium increased the percentage of M2 macrophages compared to both blank and control medium (Figure 5f). Similar changes were found in the RAW264.7 cell line (Figure S2). These data suggest that senescent FAPs promote macrophage polarization, mainly toward the M2 subtype.

## 4 DISCUSSION

Altered intercellular communication is a hallmark of aging (Lopez‐Otin et al., 2023). Senescence is a plausible disruptor of cell cross‐talk as it radically transforms the secretory phenotype of cells. In this study, we demonstrate that FAPs, a cell population that is critical for skeletal muscle tissue homeostasis, and is prone to senescence with chronological aging (Zhang et al., 2022), exhibit a significantly altered secretome when senescent. Notably, this is evidenced by both the loss of characteristic signals critical for tissue homeostasis and the gain of proinflammatory signals which, in part, drive the recruitment and M2 polarization of macrophages. These findings provide new insights into how senescent cells mediate tissue aging and new hypotheses about how senescent FAPs affect skeletal muscle composition, function, and adaptability in older organisms.

Our analysis of scRNA‐seq data from the skeletal muscle of old mice revealed distinct intercellular communication patterns between the subpopulation of senescent *p16*‐positive FAPs and macrophages compared to non‐senescent FAPs and macrophages. In particular, senescent FAPs exhibited significantly higher expression of two specific signaling molecules, *Ccl2* and *Spp1* (which encodes OPN). CCL2 is a recognized component of the SASP (Moiseeva et al., 2023; Schafer et al., 2020) and a well‐known chemokine (Chambers et al., 2021) previously reported to induce M2 macrophage polarization in human monocytes (Roca et al., 2009), partially through the interaction with its receptor, CCR2, and the downstream activation of genes associated with M2 polarization (Sierra‐Filardi et al., 2014). Consistent with these reports, cell–cell communication analyses of skeletal muscle scRNA‐seq data revealed senescent FAPs as the unique source of CCL signals and macrophages as the primary receivers, given their high expression of CCR2. Moreover, in our in vitro model, conditioned medium of senescent FAPs was highly enriched in CCL2, robustly stimulated macrophage migration, and drove M2 polarization compared to conditioned medium from control FAPs. There are in vivo and ex vivo studies demonstrating that upregulation of inflammatory pathways, inclusive of *Ccl2*, is a shared characteristic among injury‐induced senescent FAPs, satellite cells, and macrophages in young mice, which mirrors that of senescent FAPs in skeletal muscle of old mice (Moiseeva et al., 2023). These studies have further shown that genetic and pharmacological targeting of senescent cells improves muscle regeneration following injury, in part, by reducing inflammation. Together, our findings and these published studies lend support to the premise that senescent FAPs, in part through CCL2, negatively impact skeletal muscle homeostasis and regenerative capacity, and warrant further investigation.

Osteopontin is a versatile protein that, among many biological activities, mediates chemotaxis of immune cells. Similar to the CCL2‐CCR2 communication pattern, the receptor for OPN, CD44, is predominantly expressed by macrophages. Additionally, OPN levels were appreciably higher in conditioned medium of senescent compared to control FAPs, and significantly contributed to macrophage recruitment in vitro. In contrast to CCL2, OPN may not directly influence macrophage polarization, as subtypes of adipose tissue macrophages and BMDM are not altered in mice deficient for *Spp1* (Schuch et al., 2016). However, OPN has been shown to induce a senescence‐like phenotype in adipose tissue macrophages through CD44, evidenced by increased *p16*, senescence‐associated B‐galactosidase activity, proinflammatory and profibrotic behavior, and impaired efferocytosis (Sawaki et al., 2023). Of note, *Spp1* is also highly expressed in dystrophic skeletal muscle, and constitutive deletion of *Spp1* in the *mdx* mouse attenuates immune cell infiltration and fibrosis (Capote et al., 2016; Vetrone et al., 2009). The extent to which senescent FAPs‐derived OPN recruits and affects the behavior of macrophages and influences skeletal muscle structure and function in the context of aging and disease is of particular interest.

We acknowledge the inherent limitations of studying complex biological processes in vitro. In particular, we note that cells driven to senescence in vitro by either irradiation or etoposide may not perfectly phenocopy cells that undergo senescence consequent to aging in vivo. However, our comparative analysis of the transcriptional profiles suggests that core properties of senescence observed in *p16* ^*Ink4a*^‐positive senescent FAPs from old mice are reflected in the in vitro models. Even so, our conclusions related to the interplay between senescent FAPs and macrophages need to be interpreted within the context of the model and require further validation.

It is commonly reported that the SASP promotes the proliferation and M1 polarization of macrophages (Irvine et al., 2014; Kale et al., 2020; Lujambio et al., 2013). Our data suggest that the secretome of senescent FAPs, inclusive of CCL2, instead induces M2 polarization, highlighting the heterogeneity and context‐dependency of the senescence program and the SASP. It has recently been reported that M2, not M1, macrophages increase with age in human skeletal muscle (Cui et al., 2019; Cui & Ferrucci, 2020). Moreover, in old mice, the M2 macrophage subtype has been shown to promote both skeletal muscle fibrosis (Wang et al., 2015), and interestingly, the differentiation of skeletal muscle FAPs toward adipocytes (Wang et al., 2015). The extent to which the senescence‐associated shift in FAP‐macrophage communication and the increase in outgoing signals account for age‐related skeletal muscle inflammation and fibrosis is worthy of further investigation and may offer a novel therapeutic target to improve late‐life skeletal muscle health and function.

## AUTHOR CONTRIBUTIONS

Conceptualization, X.Z., N.K.L.; Investigation, X.Z., Y.E.N., L.C.S., A.A.H., W.A.H., H.L., H.H., M.L.D., S.K.; Writing, X.Z., Y.E.N., H.L., N.K.L.; Supervision, N.K.L.; Funding Acquisition, X.Z., N.K.L.

## CONFLICT OF INTEREST STATEMENT

The authors declare no competing interests.

## Supporting information


Figure S1.

Figure S2.

Table S1.

## Data Availability

RNA‐seq data of FAPs generated in this study are available at the Gene Expression Omnibus (GSE247975). scRNA‐seq data of skeletal muscle from young and old mice are publicly available at the Gene Expression Omnibus (GSE172410).
