# Supplementary figures and images for: Senescent skeletal muscle fibroadipogenic progenitors recruit and promote M2 polarization of macrophages
Source: Aging Cell. 2023 Dec 19;23(3):e14069. doi: 10.1111/acel.14069 (PMC10928562; doi:10.1111/acel.14069)

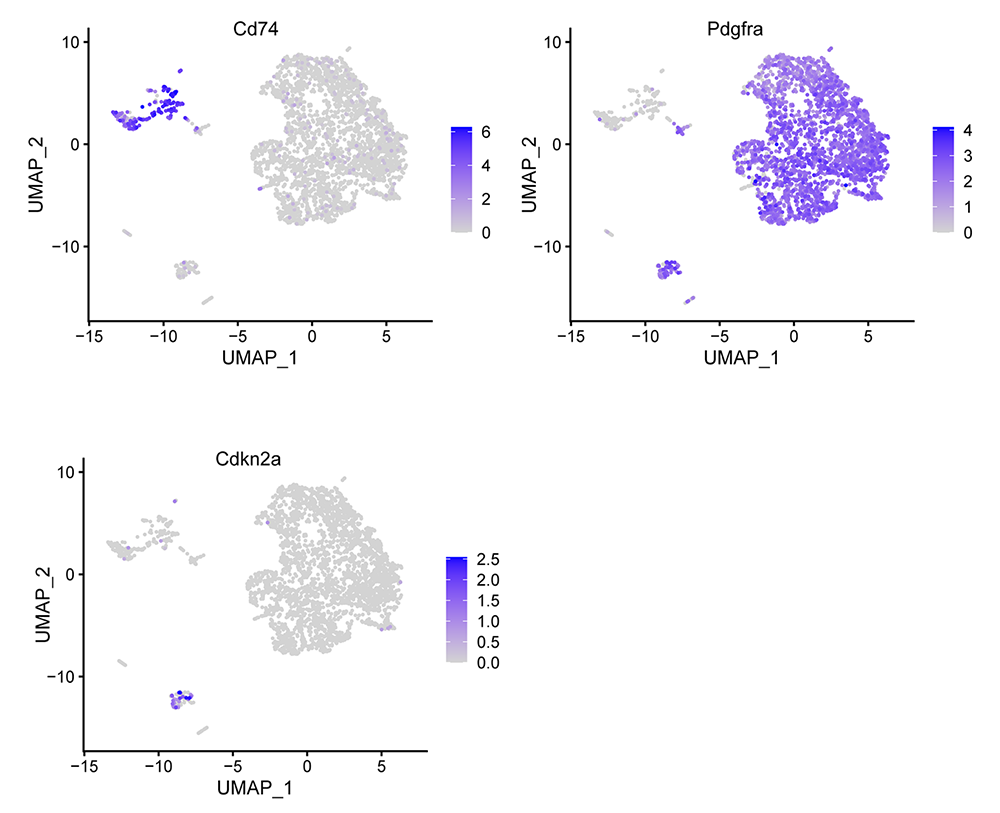

Supplement: Supplementary file 1 — Figure S1. [file ACEL-23-e14069-s002.tif]

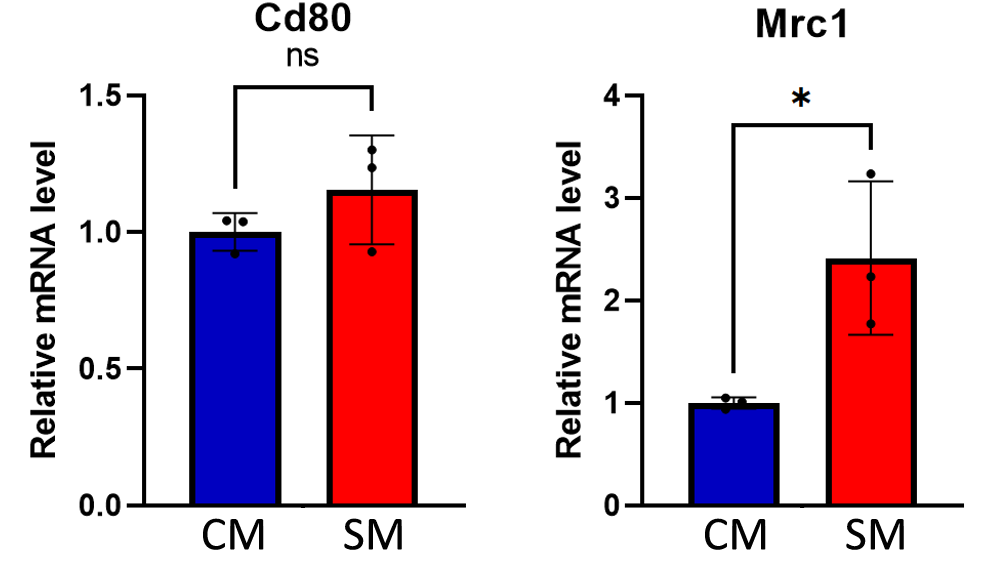

Supplement: Supplementary file 2 — Figure S2. [file ACEL-23-e14069-s001.tif]
